# Cascading effects of pre-adult survival on sexual selection

**DOI:** 10.1098/rsos.211973

**Published:** 2022-04-13

**Authors:** Hope Klug, Chelsea Langley, Elijah Reyes

**Affiliations:** ^1^ Department of Biology, Geology, and Environmental Science, University of Tennessee at Chattanooga, Chattanooga, TN, USA; ^2^ SimCenter, University of Tennessee at Chattanooga, Chattanooga, TN, USA; ^3^ Department of Biological Sciences, Simon Fraser University, Burnaby, CA, USA

**Keywords:** sexual selection, mate competition, life history, pre-adult survival, natural selection, genetic drift

## Abstract

Sexual selection influences broad-scale patterns of biodiversity. While a large body of research has investigated the effect of mate competition on sexual selection, less work has examined how pre-adult life history influences sexual selection. We used a mathematical framework to explore the influence of pre-adult survival on sexual selection. Our model suggests that pre-adult male mortality will affect the strength of sexual selection when a fixed number of adult males have an advantageous mate-acquisition trait. When a fixed number of males have an advantageous mate-acquisition trait, sexual selection is expected to increase when pre-adult mortality is relatively low. By contrast, if a fixed proportion (rather than number) of adult males have a mate-acquisition trait, pre-adult male mortality is not expected to affect the strength of sexual selection. Further, if the advantageous mating trait affects pre-adult survival, natural and sexual selection can interact to influence the overall selection on the mating trait. Given that pre-adult mortality is often shaped by natural selection, our results highlight conditions under which natural selection can have cascading effects on sexual selection.

## 1. Introduction

Sexual selection (i.e. selection that arises from fitness differences associated with non-random success in the competition for access to gametes [1]) drives patterns of biodiversity and is influenced by a range of factors [2–11] including the adult sex ratio (ratio of adult males to females in a population, ASR [9,12]), operational sex ratio (ratio of males to females prepared to mate at a given time and place, OSR [13]) [12–14], and the time spent processing consequences of mating [15]. As predicted, a large body of research has revealed that mate competition and choice and the consequences of mating (e.g. parental care) influence sexual selection [12,15–18]. Importantly, the individuals in the mating pool—and hence, the individuals that mate and enter the post-mating pool stage—are determined by the specific individuals that survive pre-adult life-history stages. Similarly, ASR and OSR are determined in part by the primary sex ratio and sex ratio at maturation, which is influenced by pre-adult survival, a trait (or collection of traits) that is frequently under strong natural selection [10,15,17,19–21]. As a result, fully understanding the operation of sexual selection necessitates that we understand the effects of pre-adult survival on subsequent sexual selection. While previous research suggests that viability selection during early life-history stages can impede subsequent selection on reproductive traits [22], we lack a clear understanding of the specific conditions under which pre-adult survival is likely to impact sexual selection.

Previous research suggests that early life history can influence mating dynamics [3,12,21,23–33]. With regard to which individuals enter the mating pool, sex differences in pre-adult survival and maturation occur in a range of animals [21,30,34,35] and can lead to a biased ASR and/or OSR. Biases in ASR and OSR can in turn affect the costs and benefits of mate competition and the direction and strength of sexual selection on phenotypic traits (reviewed in [21]). In some polygamous birds, for example, ASR is determined primarily by juvenile survival [30,36], and in the snowy plover (*Charadrius nivosus*), sex biases in juvenile survival lead to sex biases in ASR [30]. This ASR bias is associated with female-biased polygamy, suggesting that pre-adult survival can have cascading effects on mating systems [30].

In addition to influencing which individuals enter the mating pool, selection for pre-adult survival can trade off with sexually selected traits [22,37,38], and in some cases natural selection can limit the evolutionary response to sexual selection [39,40]. For example, in the bicolour damselfish (*Stegastes partitus*), larger adult males tend to produce more offspring because larger males retain a nest longer than smaller males, maintain relatively high-quality territories, and receive more eggs from females than smaller males [41]. In addition, larger males might also engage in more courtship than smaller males [41]. Thus, there are clear mating and reproductive benefits of being relatively large as an adult male. However, because larger individuals mature at a later age, a trade-off exists between large male body size and cumulative juvenile survival (i.e. males who mature later and larger are more likely to die during the juvenile stage); this trade-off in turn limits the overall selection for large male body size in the bicolour damselfish [41]. Such trade-offs between early survival and reproductive traits involved in mate competition and acquisition can depend on environmental conditions [37] and ecological interactions [38]. In the dragonfly *Pachydiplax longipennis*, a trade-off exists between pre-adult survival and the development of a sexually selected ornament, but only when predation risk is relatively high [38]. Such research suggests that early life history, which is often shaped by natural selection, can have cascading impacts on sexual selection.

Despite some previous research on the impact of pre-adult survival on sexual selection (described above), most research on sexual selection focuses primarily on mating pool dynamics (electronic supplementary material, table S1), and life-history theory and sexual-selection theory have largely been developed independently (e.g. [2,42]; but see [43]). In particular, theoretical models of sexual selection tend to focus primarily on adult life history (discussed in [6]). While biologists studying sexual selection would probably uniformly agree that pre-adult survival affects sexual selection, we lack *a priori* hypotheses regarding the conditions under which pre-adult survival will affect sexual selection. To identify the conditions under which pre-adult life history is expected to affect sexual selection, we use a mathematical framework to explore the effect of pre-adult survival on sexual selection.

## 2. Methods

**Figure 1.**
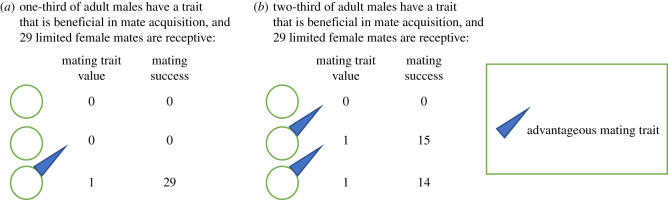
An illustration of mate acquisition and sexual selection in the model. Adult males either have or lack a binary sexually selected trait that allows them to acquire and reproduce with limited female mates (*a*,*b*). Only males that have the mating trait can acquire mates. The advantageous mating trait is represented as a blue triangle in the figure. Males with the advantageous trait are assigned a mating-trait value of 1 to indicate that they have the trait; males that lack the trait are assigned a value of 0 to indicate that they do not have the mating trait. If we imagine, for example, that there are 29 receptive female mates and we assume that all males with the advantageous mating trait have equal likelihood of acquiring female mates, mating success for each male is calculated by distributing the 29 female mates as equitably as possible among males who possess the mating trait while maintaining an integer value for mating success (*a*,*b*). In some cases, because mate number must be an integer (i.e. because it is impossible to have a fraction of a female mate), there will be variation in mating success among males with the mating trait (*b*).

We use toy models (deliberately simple models [44]) to explore the effect of pre-adult survival on sexual selection. We intentionally focus on relatively simple scenarios to provide a set of foundational *a priori* hypotheses of how pre-adult survival can influence sexual selection, thereby providing testable hypotheses of the cascading effect of pre-adult survival on sexual selection. We focus on pre-adult mortality, which we assume applies to the total mortality experienced during pre-adult life-history stages. The results of our model could also apply to mortality experienced in any given pre-adult life-history stage. Pre-adult mortality can occur due to chance and/or because some individuals have one or more phenotypic traits that allow them to survive better than those who lack such traits. Variation in pre-adult survival in our model could be thought of as stemming from either chance or natural selection (i.e. our model is consistent with genetic drift or natural selection influencing pre-adult survival). In the electronic supplementary material, to illustrate specifically the link between natural selection on pre-adult survival and sexual selection on a male trait, we provide an example of how natural selection could shape a pre-adult survival trait (electronic supplementary material, figures S1 and S2). In the model, we assume that males can mate multiply, whereas females mate only once during a given reproductive episode, such that mate availability only affects male fitness. Males either have or lack a trait that is beneficial in mate acquisition and fertilization success, and only males who have this trait can mate (i.e. this trait is binary, expressed in males, and confers a mating and reproductive advantage; figure 1). This trait could be thought of as any discrete male trait that is preferred by females during mate choice or that is beneficial in male–male competition that leads to increased mating and fertilization success (figure 1).

In the model, we considered an initial population with equal numbers of pre-adult males and females (discussed below). We then quantified the relationship between pre-adult male mortality and the strength of sexual selection on the male mating trait across scenarios in which (i) pre-adult survival was either different or equal between the sexes and (ii) either a fixed proportion or fixed number of males had the mating trait. We specifically considered the following scenarios: males and females have equal pre-adult mortality and a fixed proportion of surviving adult males have the mating trait (Scenario 1); males and females have different pre-adult mortality and a fixed proportion of surviving adult males have the mating trait (Scenario 2); males and females have equal pre-adult mortality and a fixed number (rather than a fixed proportion) of surviving adult males have the mating trait (Scenario 3); males and females have different pre-adult mortality and a fixed number of surviving adult males have the mating trait (Scenario 4). We chose these scenarios as they represent a broad range of possible survival and male-trait distribution scenarios.

We considered the following values of pre-adult mortality and male mating-trait distribution: male and female pre-adult mortality is 10%, 40% or 70% across three levels of male-trait distribution (1/3, 2/3 or 8/9 of males that survive to adulthood have the mating trait) (Scenario 1); female pre-adult mortality is 10% and male pre-adult mortality is 10%, 40% or 70% across three levels of male-trait distribution (1/3, 2/3 or 8/9 of males who survive to adulthood have the mating trait) (Scenario 2); male and female pre-adult mortality is 10%, 40% or 70% across three levels of male-trait distribution (3, 6, or 9 of males that survive to adulthood have the mating trait) (Scenario 3); and female pre-adult mortality is 10% and male pre-adult mortality is 10%, 40% or 70% across three levels of male-trait distribution (3, 6 or 9 of males that survive to adulthood have the mating trait) (Scenario 4). The parameters were chosen as they reflect a broad range of mortalities and male-trait distributions and therefore probably apply to a range of systems. As mentioned above, variation in pre-adult survival could be associated with genetic drift and/or natural selection, and to illustrate how natural selection could impact pre-adult mortality, we provide a scenario in which natural selection acts on a pre-adult survival trait in the electronic supplementary material (see also electronic supplementary material, figures S1 and S2). Likewise, the adult male mating trait distributions outlined above could be reflective of genetic drift or natural selection on the male mating trait, and whether natural selection affects the mating trait will depend on the initial frequency of the mating trait among pre-adult males (discussed further below).

For each level of pre-adult mortality and male-trait distribution associated with each scenario, we assumed that each population initially consisted of 30 pre-adult males and 30 pre-adult females. Individuals who survived the pre-adult stage were assumed to successfully mature and have the potential to reproduce. For each case, only males with the mating trait could receive one or more female mates (figure 1). As mentioned above, we assumed that males could mate multiply whereas females mate once. To determine the mating success of each male in each case, female mates were assigned as equitably as possible among males with the mating trait while restricting mating success to an integer value (see also [45]; figure 1). Thus, mating success is determined by a male's trait value, although some stochasticity in mating success arises due to the fact that mate number is restricted to an integer value [45]. We do not assume any other source of stochasticity in the model. For simplicity, we assume that mating success is directly proportional to fertilization success (i.e. we do not consider variation in female fecundity and assume that all matings result in equally successful fertilization). As a result, sexual selection arises from variation in mating success in our model. For each case, we quantified the strength of sexual selection by calculating the sexual selection differential associated with the mating trait [46]. The sexual selection differential (*s*_sex_) quantifies the strength of sexual selection on the mating trait and is proportional to the response to sexual selection expected if there is heritable variation associated with the trait and if mating success is correlated with reproductive success. The sexual selection differential was calculated as follows:

2.1 $$s_{\mathrm{sex}}=\mathrm{cov}(x_{\mathrm{male}},W_{\mathrm{mat}}),$$

where *x*_male_ is the male mating trait value and *W*_mat_ is relative mating success [46]. Only males who survived to adulthood were included in the calculation of the sexual selection differential. That is, males who died before maturation and were, therefore, unable to compete for mates were excluded from the calculation of sexual selection strength. This allowed us to explore the cascading effect of pre-adult survival on sexual selection without confounding natural and sexual selection. All numerical analyses were also performed using the standardized sexual selection differential [46] and the patterns were qualitatively identical (electronic supplementary material, figure S3).

In addition, because a fixed number or proportion of males have the mating trait as adults (Scenarios 1–4 above), pre-adult survival will in some cases be non-randomly associated with the mating trait. For example, if only males with the mating trait survive to adulthood, there will be positive natural selection associated with the mating trait. Such a pattern might be expected if some males are ‘better’ than others and are both more likely to survive the pre-adult stage and have an advantageous mating trait. By contrast, having the mating trait could be associated with increased pre-adult mortality (e.g. if there is a trade-off between investment in the development of the mating trait and pre-adult survival), in which case natural selection would select against the mating trait. In other cases, males who have the mating trait might be equally likely to survive the pre-adult stage as males who lack the mating trait, in which case there would be no natural selection associated with the mating trait. To explore how natural selection can be associated with the mating trait, we calculated the minimum and maximum possible strength of natural selection (i.e. the minimum and maximum selection differential) that could be associated with the adult mating trait frequencies outlined in Scenarios 1–4 above. The selection differential, *s*_nat_, was calculated as follows:

2.2 $$s_{\mathrm{nat}}\;=\;\mathrm{cov}(x_{\mathrm{male}},W_{\mathrm{surv}}),$$

where *x*_male_ is the male mating trait value and *W*_surv_ is relative pre-adult survival success [46]. We then used these analyses to explore whether the level of male pre-adult survival and the abundance of the mating trait among adult males was related to natural selection on the mating trait. All analyses were also performed using the standardized selection differential [46] and the patterns were qualitatively identical (electronic supplementary material, figure S4).

All data are available at https://doi.org/10.5061/dryad.v41ns1rzk.

## 3. Results

### 3.1. Pre-adult mortality can have cascading effects on sexual selection

**Figure 2.**
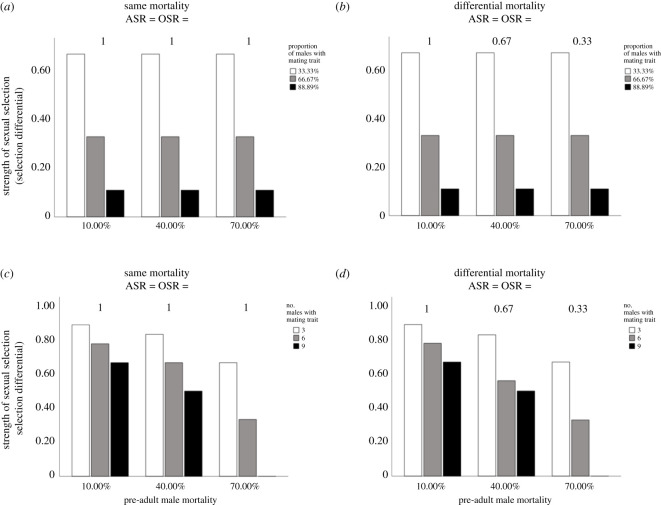
Effects of pre-adult mortality on sexual selection. (*a*) When a fixed proportion of males have an advantageous mating trait, equal pre-adult mortality among males and females creates an unbiased ASR and OSR (i.e. ASR = OSR = 1). The strength of sexual selection is greater when a smaller proportion of males have the mating trait but pre-adult male mortality does not influence the strength of sexual selection (i.e. the sexual selection differential on the male mating trait). (*b*) When a fixed proportion of males have an advantageous mating trait, sex differences in pre-adult mortality lead to a biased ASR and OSR (i.e. ASR and OSR ≠ 1). The strength of sexual selection is greater when a smaller proportion of males have the mating trait but pre-adult male mortality does not influence the strength of sexual selection. (*c*,*d*) If a fixed number of adult males (rather than a fixed proportion) have the advantageous mating trait, increasing pre-adult male mortality is related to a decrease in the strength of sexual selection on the preferred trait when (*c*) males and females have the same pre-adult mortality and (*d*) males and females differ in pre-adult mortality.

Pre-adult mortality will in some—but not all—cases influence sexual selection. When males and females had equal pre-adult mortality and a fixed proportion of adult males had the mating trait (Scenario 1), ASR and OSR were equal to one across pre-adult mortality levels, and the proportion of males with the mating trait, but not pre-adult mortality, determined the strength of sexual selection (figure 2*a*). Sex differences in pre-adult mortality, a pattern documented in a range of animals [30,47], led to sex biases in ASR and OSR (figure 2*b*). However, when a fixed proportion of males had the mating trait, the proportion of males with the mating trait, but not pre-adult male mortality, determined the strength of sexual selection (Scenario 2; figure 2*b*). Specifically, the strength of sexual selection increased when a smaller proportion of males had the mating trait. This pattern occurs because there is greater mate monopolization by the few males (i.e. the small proportion of males) with the preferred trait. That is, when a relatively small proportion of males had the mating trait, a relatively large proportion of males remained unmated and those males with the advantageous trait had relatively high mating success, which led to high non-random variation in mating success.

By contrast, if a fixed number, rather than a fixed proportion, of adult males had the mating trait, both pre-adult male survival and the number of males with the mating trait affected the strength of sexual selection (Scenarios 3 and 4; figure 2*c,d*). Specifically, sexual selection strength increased when pre-adult male mortality was relatively low and when a relatively small number of males had the preferred trait (figure 2*c,d*). This pattern was consistent regardless of whether males and females had the same or different pre-adult survival (figure 2*c,d*). In particular, when relatively few males (three in our scenarios) had the advantageous mating trait and when pre-adult male mortality was low (i.e. when many males survived the pre-adult stage), the strength of sexual selection was greatest. This pattern occurs because relatively few males monopolize all female mates under these conditions. Specifically, when a large number of males survive to adulthood but few have the advantageous trait, many males remain unmated; the few that do have the advantageous trait are then able to acquire and monopolize the female mates in the population and as a result have relatively high mating success. Because mating success is then skewed heavily toward the few males with the advantageous mating trait, sexual selection is relatively strong. By contrast, if many males die before reaching adulthood and a fixed number of males have the preferred trait, there is less non-random variation in mating success, which reduces the strength of sexual selection.

### 3.2. Natural selection can influence selection on the mating trait during the pre-adult stage

**Figure 3.**
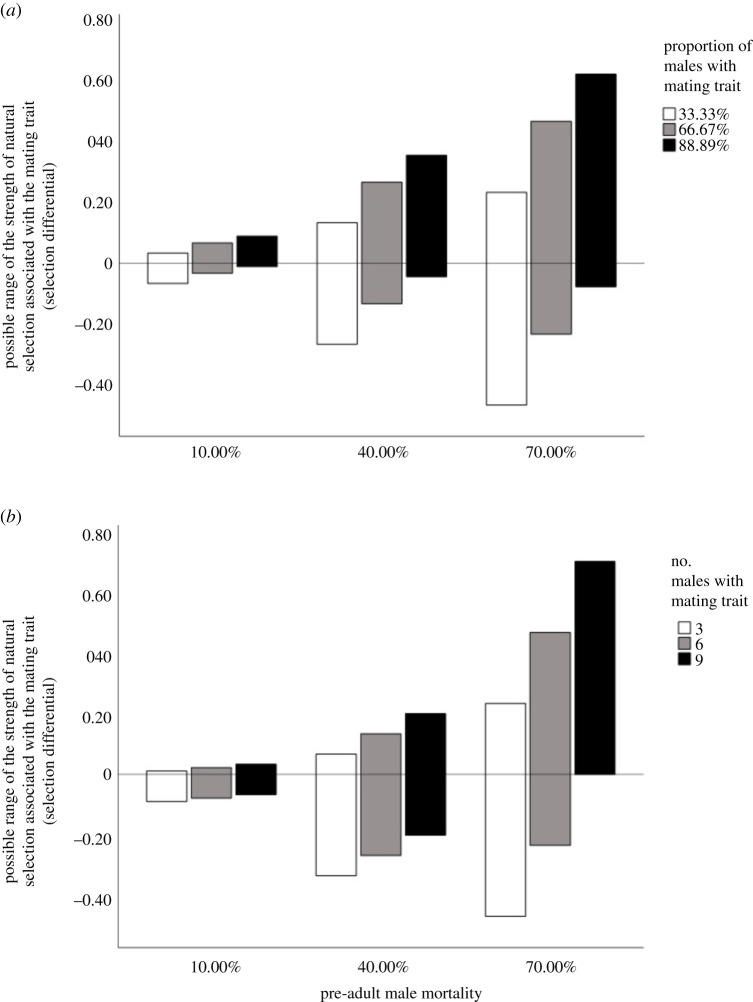
Range in the strength of natural selection that could be associated with the mating trait. Here, we depict the range (minimum to maximum) in the strength of natural selection that could potentially be associated with the mating trait when (*a*) a fixed proportion of males have an advantageous mating trait and (*b*) a fixed number of males have an advantageous mating trait. Bars represent the range of possible selection differential values for each level of pre-adult mortality and each level of adult male mating trait abundance.

In our scenarios, there is potential for natural selection to influence the mating trait during the pre-adult stage. If individuals with the mating trait are more or less likely to survive the pre-adult stage relative to individuals who lack the mating trait, natural selection will favour or disfavour the mating trait (figure 3). The maximum possible positive natural selection that favours the mating trait will increase as pre-adult male mortality increases (figure 3*a,b*) and as the proportion (figure 3*a*) or number (figure 3*b*) of adult males with the mating trait increases. Likewise, when a fixed proportion of adult males have the mating trait, natural selection against the mating trait (i.e. negative selection) has the potential to be strongest when male pre-adult mortality is relatively high and when a relatively small proportion of males have the mating trait (figure 3*a*). When a fixed number (rather than a fixed proportion) of adult males have the mating trait, pre-adult male mortality and the number of males with the mating trait will interact to determine the maximum possible strength of natural selection that can oppose the mating trait (figure 3*b*). Specifically, when a fixed number of males have the mating trait, natural selection against the mating trait has the potential to be strongest when pre-adult male mortality is relatively high and relatively few (i.e. only three) adult males have the mating trait (unshaded bars, figure 3*b*). When a greater number (i.e. six or nine) of adult males have the mating trait, natural selection against the mating trait has the potential to be strongest when pre-adult male mortality is intermediate (grey and black bars, figure 3*b*).

Importantly, natural selection associated with the mating trait can act in the same direction as sexual selection (i.e. both natural and sexual selection can be positive, figures 2 and 3), which would facilitate the evolution of the mating trait, or natural sexual can oppose sexual selection (i.e. natural selection can be negative when sexual selection is positive, figures 2 and 3), which would be expected to limit the overall evolutionary response to selection associated with the mating trait. In some cases, there is potential for there to be no natural selection associated with the mating trait (figure 3). In all cases considered in our model, the overall selection on the mating trait will be positive or zero (figures 2 and 3); however, the relative importance of natural and sexual selection associated with the mating trait will depend on pre-adult male mortality, whether a fixed number or proportion of adult males have the mating trait, and the frequency of the mating trait among pre-adult males in the population. Regardless of any natural selection associated with the mating trait (figure 3), pre-adult survival will have cascading effects on sexual selection when a fixed number of adult males have the mating trait during adulthood (figure 2).

In summary, both sexual (figure 2) and natural (figure 3) selection can act on the male mating trait. Regardless of any natural selection that occurs with respect to the mating trait during the pre-adult period (figure 3), the overall selection on the mating trait will be positive or zero in our scenarios (figures 2 and 3). Further, regardless of whether there is natural selection associated with a survival trait (electronic supplementary material, figure S2) or the mating trait (figure 3), pre-adult survival can have cascading effects on the strength of sexual selection (figure 2).

## 4. Discussion

Pre-adult mortality impacts the composition of the mating pool and in some cases the strength of sexual selection. Regardless of whether male and female pre-adult mortality values are equal or different, pre-adult mortality will influence sexual selection when a fixed number of surviving adult males have an advantageous mating trait. As pre-adult male mortality increases, and as the number of males with the mating trait increases, the strength of sexual selection on an advantageous mating trait will decrease. This pattern occurs because mate monopolization, which determines the strength of sexual selection [13,48], will be greatest when a relatively large number of males are present but only a few males have the advantageous trait, a situation that is expected to occur when pre-adult male mortality and male-trait abundance are both relatively low. In other words, if pre-adult male mortality is high, relatively few males will survive to adulthood; if a fixed number of those surviving males have an advantageous mating trait there will be less non-random variation in mating success relative to the scenario in which pre-adult mortality is low and many males survive to adulthood. Contrastingly, pre-adult mortality will not influence sexual selection when a fixed proportion of males have an advantageous mating trait. Such a pattern occurs because regardless of male abundance when a fixed proportion of males have the preferred trait, mate monopolization remains invariant across levels of pre-adult male mortality.

Previous empirical research has found that strong pre-adult viability selection can limit or weaken subsequent sexual selection on adult traits [22,37–39,41]. In some cases, naturally selected traits that increase pre-adult survival trade off with sexually selected traits that are expressed later in life [22,38,39,41]. In our model, such a trade-off between natural and sexual selection is possible and can lead to natural selection opposing the mating trait, which can in turn weaken the overall strength of selection for the mating trait (figure 3). Importantly, though, pre-adult survival does not necessarily trade off with the sexually selected mating trait in our model. For the scenarios in our model, the adult male mating trait frequencies considered could be associated with negative, positive, or no natural selection on the mating trait (figure 3). The direction and strength of natural selection associated with the mating trait will depend on pre-adult mortality, whether a fixed number or proportion of adult males have the mating trait, and the abundance of the male mating trait among pre-adult males.

In general, our findings suggest that both natural and sexual selection have the potential to act on an advantageous male mating trait. Regardless of the strength and direction of natural selection associated with the mating trait, pre-adult mortality can influence sexual selection, and when a fixed number of males have an advantageous mating trait, high pre-adult male mortality will lead to weaker sexual selection. High pre-adult mortality could be associated with natural selection on a survival trait (electronic supplementary material, figures S1 and S2), but it could also be associated with genetic drift. The above patterns provide a set of *a priori* predictions regarding the effect of pre-adult survival on sexual selection (table 1). Our finding that pre-adult survival can in some cases influence sexual selection is consistent with previous empirical research demonstrating that pre-adult life history influences sexual selection [3,11,15,19,21,25–28,30,32,36,49–51]. More generally, our results suggest that fully understanding mate acquisition requires that we consider the impact of pre-adult mortality on sexual selection, particularly when a fixed number of adult males have an advantageous mating trait. Pre-adult mortality that is associated with chance events and/or natural selection can have cascading effects on subsequent sexual selection.

**Table 1.** Predicted effects of pre-adult male mortality on the strength of sexual selection on a male trait. Pre-adult mortality could be associated with natural selection (if individuals have traits that influence pre-adult survival) or with chance events.

scenario	predicted effect on sexual selection
a fixed proportion of surviving adult males have a trait that provides an advantage in relation to mate acquisition	Equal pre-adult mortality among males and females creates an unbiased ASR and OSR. Pre-adult mortality is not expected to influence the strength of sexual selection on mate-acquisition traits.
	Sex differences in pre-adult mortality will lead to a biased ASR and OSR. Pre-adult mortality is not expected to influence the strength of sexual selection on mate-acquisition traits.
a fixed number of surviving adult males have a trait that provides an advantage in relation to mate acquisition	Equal pre-adult mortality among males and females creates an unbiased ASR and OSR. Increasing male pre-adult mortality is expected to decrease the strength of sexual selection on mate-acquisition traits.
	Sex differences in pre-adult mortality will lead to a biased ASR and OSR. Increasing male pre-adult mortality is expected to decrease the strength of sexual selection on mate-acquisition traits.

## Data Availability

Data available from the Dryad Digital Repository: https://doi.org/10.5061/dryad.v41ns1rzk [52]. The data are also provided in electronic supplementary material [53].
